# 

**Published:** 1890-04

**Authors:** 


CONTENTS

VOL. 37 No. 4.

| | PAGE. |
|---|---|
| Special | 73 |
| Looking Forward | 73 |
| How Long to Sleep | 77 |
| A Doctor's "Don'ts" | 78 |
| "Lay Not Thy Hand to the Plowing Beast" | 79 |
| Eczema | 80 |
| The Fear of Death | 81 |
| Anxiety | 82 |
| Digestion | 82 |
| What's in a Dream | 83 |
| A Test of Death | 83 |
| A Sanitary Wash-House | 84 |
| Fasting | 85 |
| The Danger of Tea Drinking | 86 |
| The Invisible World | 86 |
| Ideas of a Future Life | 87 |
| The Art of Prolonging Life | 88 |
| Mummified Cats | 88 |
| The Nose | 89 |
| Concerning Food | 90 |
| Why the Sea is Green | 90 |
| Sleep | 91 |
| A Wonderful Flower | 91 |
| Household | 92 |
| Miscellaneous | 93 |
| Literary | 95 |

INDEX TO ADVERTISEMENTS OF HALF A PAGE AND OVER.

| | PAGE. |
|---|---|
| An Unprecedented Offer | I |
| Celluloid | II |
| Hollow Suppositories | III |
| Christ Before Pilate | IV |
| Lactopeptine | V |
| Goldsmith's Pianos | V |
| E. C. Morris & Co | VI |
| Dennin's Certain Cure | VII |
| Hospital Remedy Co | IX |
| Herb Cure Co | X |

